# A new species of *Cenopalpus* Pritchard & Baker (Acari: Tenuipalpidae) from Japan, with ontogeny of chaetotaxy and a key to the world species

**DOI:** 10.7717/peerj.9081

**Published:** 2020-04-27

**Authors:** Mohamed W. Negm, Edward A. Ueckermann, Tetsuo Gotoh

**Affiliations:** 1 Laboratory of Applied Entomology and Zoology, Faculty of Agriculture, Ibaraki University, Ami, Ibaraki, Japan; 2 Department of Plant Protection, Faculty of Agriculture, Assiut University, Assiut, Egypt; 3 Unit for Environmental Sciences and Management, North-West University, Potchefstroom, South Africa; 4 Faculty of Economics, Ryutsu Keizai University, Ryugasaki, Ibaraki, Japan

**Keywords:** Acarology, Systematics, Acari, Trombidiformes, Prostigmata, Phytophagous, Classification, Pest

## Abstract

A new species of flat mite, *Cenopalpus umbellatus* sp. nov. (Acari: Trombidiformes: Tenuipalpidae) is described and illustrated based on females, males, deutonymphs, protonymphs and larvae. The morphological ontogeny in idiosomal and leg chaetotaxy is briefly described for all stages. Mite specimens were collected from the leaves of *Rhaphiolepis indica* var. *umbellata* Makino (Rosaceae), an evergreen shrub native to Japan. An identification key to the world species of *Cenopalpus* is also provided.

## Introduction

Mites of the family Tenuipalpidae Berlese, 1913 (Acari: Trombidiformes) are harmful pests to a wide range of plants (Jeppson, Keifer & Baker, 1975; Mesa et al., 2009). The genus *Cenopalpus* Pritchard & Baker, 1958, currently contains 70 species (including the present new species), mostly described from Palearctic and Afrotropical ecozones (Table 1). Mesa et al. (2009) listed the genus *Cenopalpus* with 60 species, assigning the two species, *salignae* (Meyer, 1979) and *thelycraniae* (Livschitz & Mitrofanov, 1967), under *Brevipalpus*. Later, Saccaggi et al. (2017) cited *B. salignae* in the genus *Cenopalpus*, however, the Russian species (*B. thelycraniae*) was already transferred to *Cenopalpus* by Mitrofanov & Strunkova (1979). Also, *C. iqbali* Iqbal, Akbar & Ali, 2007, was not included in Mesa et al. (2009).

**Table 1:** List of *Cenopalpus* mites of the world (70 species)*.

	Species	Country
1	*abaii* Khosrowshahi & Arbabi, 1997	Iran
2	*adventicius* Ueckermann & Ripka, 2015	Hungary
3	*aratus* Chaudhri, 1971	Pakistan
4	*arbuti* Hatzinikolis & Emmanouel, 1987	Greece
5	*bagdasariani* (Livschitz & Mitrofanov, 1970)	Tajikistan
6	*bakeri* Düzgünes, 1967	Turkey
7	*brachypalpus* Hatzinikolis, Panou & Papadoulis, 1999b	Greece
8	*capacis* Chaudhri, 1971	Pakistan
9	*capensis* (Meyer, 1979)	South Africa
10	*carpini* (Livschitz & Mitrofanov, 1967)	Ukraine
11	*chitraliensis* Akbar & Chaudhri, 1985	Pakistan
12	*crataegi* Dosse, 1971	Iran
13	*creticus* Hatzinilkolis, Papadoulis & Panou, 1999a	Greece
14	*cumanicus* Ueckermann & Ripka, 2015	Hungary
15	*dignus* Akbar & Chaudhri, 1985	Pakistan
16	*eriobotryi* Hatzinikolis, 1969	Greece
17	*evini* Khosrowshahi, 1991	Iran
18	*favosus* Chaudhri, 1971	Pakistan
19	*halperini* Castagnoli, 1987	Israel
20	*haqii* Akbar & Chaudhri, 1985	Pakistan
21	*hederae* Papaioannou-Souliotis, 1986	Greece
22	*homalos* Akbar & Chaudhri, 1985	Pakistan
23	*iqbali* Iqbal, Akbar & Ali, 2007	Pakistan
24	*irani* Dosse, 1971	Iran
25	*japonicus* Hasan, Akbar & Khalid, 2001	Pakistan
26	*khosrowshahii* Khanjani et al., 2012	Iran
27	*kritos* Hasan et al., 2004	Pakistan
28	*lanceolatisetae* (Attiah, 1956)	Egypt
29	*limbatus* Akbar & Chaudhri, 1985	Pakistan
30	*lineola* (Canestrini & Fanzago, 1876)	Italy
31	*longirostris* (Livschitz & Mitrofanov, 1967)	Ukraine
32	*mespili* (Livschitz & Mitrofanov, 1967)	Ukraine
33	*meyerae* Khosrowshahi, 1991	Iran
34	*mughalii* Akbar & Aheer, 1990	Pakistan
35	*musai* Dosse, 1975	Lebanon
36	*natalensis* (Lawrence, 1943)	South Africa
37	*naupakticus* Hatzinikolis, Panou & Papadoulis, 1999b	Greece
38	*officinalis* Papaioannou-Souliotis, 1986	Greece
39	*oleunus* (Meyer, 1979)	South Africa
40	*orakiensis* Akbar & Chaudhri, 1985	Pakistan
41	*pegazzanoae* Castagnoli, 1987	Italy
42	*pennatisetis* (Wainstein, 1958)	Kazakhistan
43	*picitilis* Chaudhri, 1971	Pakistan
44	*piger* Wainstein, 1960	Kazakhistan
45	*pistaciae* Hatzinilkolis, Papadoulis & Panou, 1999a	Greece
46	*platani* (Livschitz & Mitrofanov, 1967)	Georgia
47	*populi* (Livschitz & Mitrofanov, 1967)	Georgia
48	*pritchardi* Düzgünes, 1967	Turkey
49	*prunusi* Khanjani et al., 2012	Iran
50	*pseudospinosus* (Livschitz & Mitrofanov, 1967)	Ukranie
51	*pterinus* Pritchard & Baker, 1958	Spain
52	*pulcher* (Canestrini & Fanzago, 1876)	Italy
53	*quadricornis* (Livschitz & Mitrofanov, 1967)	Armenia
54	*quercusi* Khanjani et al., 2012	Iran
55	*ramus* Manson, 1963	Pakistan
56	*ruber* Wainstein, 1960	Tajikistan
57	*rubusi* Khanjani et al., 2012	Iran
58	*salignae* (Meyer, 1979)	South Africa
59	*saryabiensis* Akbar & Chaudhri, 1985	Pakistan
60	*scoopsetus* Hatzinikolis & Papadoulis, 1999	Greece
61	*spinosus* (Donnadieu, 1875)	France
62	*sunniensis* Hasan et al., 2004	Pakistan
63	*tamarixi (Nassar & Kandeel)*—Zaher (1984)	Egypt
64	*taygeticus* Hatzinikolis, Panou & Papadoulis, 1999b	Greece
65	*thelycraniae* (Livschitz & Mitrofanov, 1967)	Ukraine
66	*umbellatus* sp. nov. Negm, Ueckermann & Gotoh	Japan
67	*viniferus* Hatzinikolis, Papadoulis & Kapaxidi, 2001	Greece
68	*virgulatus* Akbar & Chaudhri, 1985	Pakistan
69	*wainsteini* (Livschitz & Mitrofanov, 1967)	Ukraine
70	*xini* Ma & Li, 1984	China

**Note:**

* Synonymy. (1) *Cenopalpus fewstrii* Zaher & Yousef, 1969 (= *C. wainsteini* (Livschitz & Mitrofanov, 1967))—Hatzinikolis & Emmanouel (1987). (2) *Cenopalpus kalandadzei* (Reck, 1951) (= *C. lineola* (Canestrini & Fanzago, 1876))—Hatzinikolis & Emmanouel (1987). (3) *Brevipalpus asyntactus* Baker & Pritchard, 1952 (= *C. lineola*)—Mesa et al. (2009).

In Japan, comparing to spider mites (Tetranychidae), few studies have been done on the taxonomy of tenuipalpid mites. It is expected that several localities are most likely to hold undiscovered species. Ehara & Gotoh (2009) listed 14 species of flat mites from Japan, belonging to the genera *Aegyptobia* Sayed, *Brevipalpus* Donnadieu, *Cenopalpus*, *Dolichotetranychus* Sayed, *Pentamerismus* McGregor and *Tenuipalpus* Donnadieu, with only one species of *Cenopalpus* (*C. lineola*; Table 2). Therefore, the present work aimed to increase our knowledge about the tenuipalpid mite fauna in Japan through describing a new species of *Cenopalpus*. Since immature stages of mites can provide valuable information for better mite systematics, we have described all stages of the new species, with remarks on their ontogenetic changes. Also, an identification key to the world species of *Cenopalpus* is provided.

**Table 2:** List of tenuipalpid mites known from Japan.

Species	Reference
*Aegyptobia arenaria* Ehara, 1982	Ehara (1982)
*Brevipalpus californicus* (Banks, 1904)	Ehara (1962)
*B. lewisi* McGregor, 1949	Ehara (1956b)
*B. obovatus* Donnadieu, 1875^a^	Ehara (1956a)
*B. phoenicis* (Geijskes, 1939)	Ehara (1966)
*B. russulus* (Boisduval, 1867)	Ehara (1968)
*Cenopalpus lineola* (Canestrini & Fanzago, 1876)	Ehara (1966)
*C. umbellatus* sp. nov. Negm, Ueckermann & Gotoh	Present study
*Dolichotetranychus floridanus* (Banks, 1900)	Baker & Pritchard (1956)
*D. zoysiae* Ehara, 2004	Ehara (2004)
*Pentamerismus oregonensis* McGregor, 1949	Ehara (1962)
*P. taxi* (Haller, 1877)	Ehara (1962)
*Tenuipalpus boninensis* Ehara, 1982	Ehara (1982)
*T. pacificus* Baker, 1945	Ehara & Ohkubo (1992)
*T. zhizhilashviliae* Reck, 1953^b^	Ehara (1956b)

**Notes:**

a *Brevipalpus obovatus* (Donnadieu, 1875) was firstly reported in Japan from its synonym *T. inornatus* (Banks, 1912) by Ehara (1956a).

b *T. zhizhilashviliae* (Reck, 1953) was reported from its synonym *T. japonicus* (Nishio, 1956) by Ehara, 1956b).

## Materials and Methods

Mite collection, examination and slide preparations were conducted as previously described in Negm & Gotoh (2019). Measurements (in micrometres) were done using the imaging software Sensiv Measure^®^ ver. 2.6.0 and were presented for the holotype specimen then followed by the range for paratypes in parentheses. The terminology and abbreviations used in the description of the new species follows that of Lindquist (1985) and Mesa et al. (2009). Leg chaetotaxy is adapted from Lindquist (1985) and Seeman & Beard (2011). Several taxonomic keys to *Cenopalpus* species have been used in the present study, mostly regional (Wainstein, 1960 (Kazakhstan); Livschitz & Mitrofanov, 1967 (USSR); Zaher & Yousef, 1969, Zaher, 1984 (Egypt); Meyer, 1979 (World); Akbar & Chaudhri, 1985 (Pakistan); Hatzinikolis & Emmanouel, 1987; Hatzinilkolis, Papadoulis & Panou, 1999a; Hatzinikolis, Panou & Papadoulis, 1999b (Greece); Khosrowshahi & Arbabi, 1997; Khanjani et al., 2012 (Iran); Çobanoğlu, Ueckermann & Sağlam, 2016; Çobanoğlu, Erdoğan & Kılıç, 2019 (Turkey)).

Nomenclatural Acts. The electronic version of this article in Portable Document Format will represent a published work according to the International Commission on Zoological Nomenclature (ICZN), and hence the new names contained in the electronic version are effectively published under that Code from the electronic edition alone. This published work and the nomenclatural acts it contains have been registered in ZooBank the online registration system for the ICZN. The ZooBank Life Science Identifiers (LSIDs) can be resolved and the associated information viewed through any standard web browser by appending the LSID to the prefix http://zoobank.org/. The LSID for this publication is: urn:lsid:zoobank.org:pub:268B04C7-028B-4C03-8F6C-930035941B89, and the LSID for the new species, *Cenopalpus umbellatus* is urn:lsid:zoobank.org:act:957E754C-A7F0-4081-A814-48115D276F76. The online version of this work is archived and available from the following digital repositories: PeerJ, PubMed Central and CLOCKSS.

## Results

Family Tenuipalpidae Berlese, 1913

*Cenopalpus* Pritchard & Baker, 1958

***Cenopalpus umbellatus* sp. nov.**

[Japanese name: Sharimbai-himehadani]

(Figs. 1–10)

**Figure 1:**
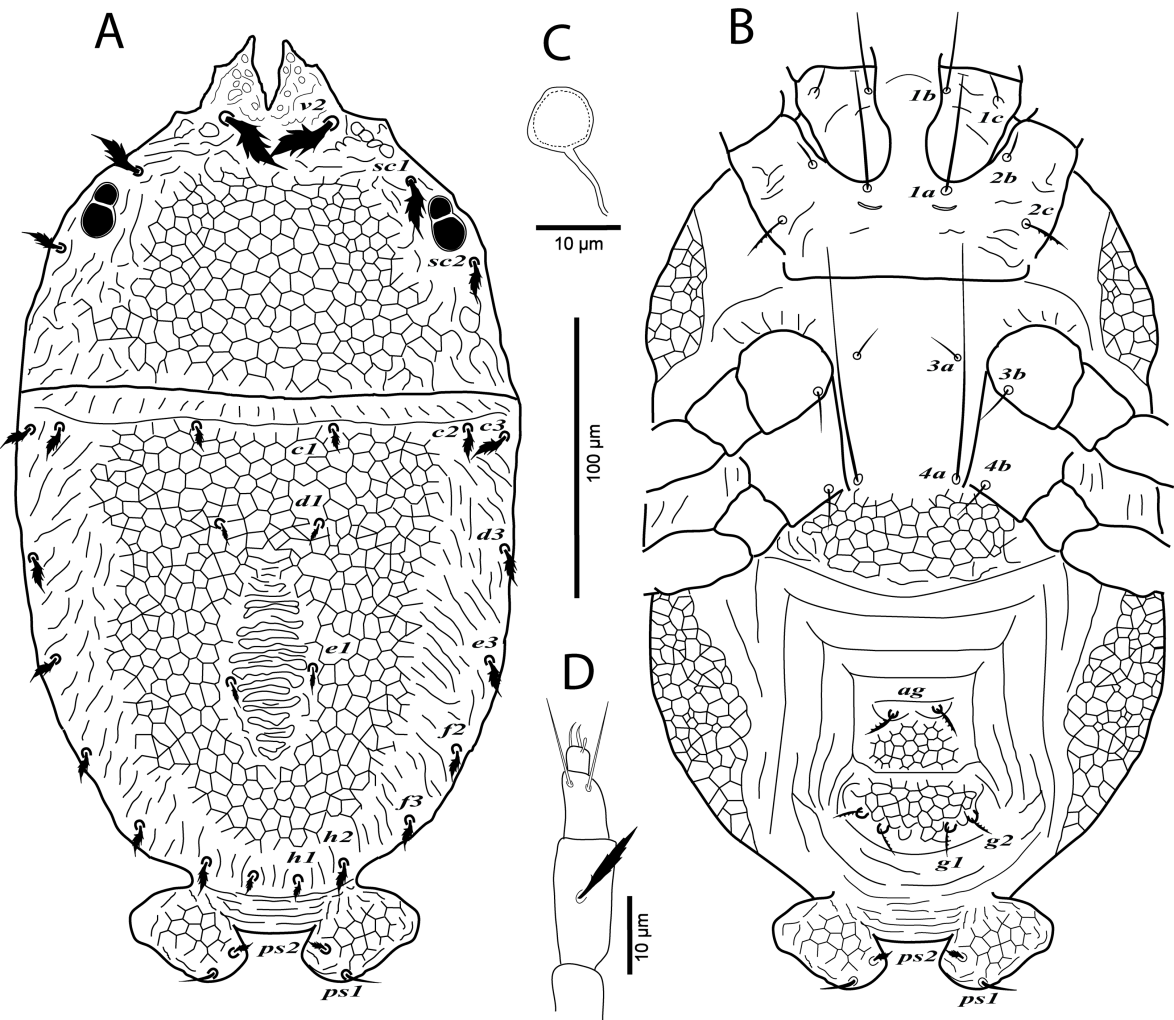
*Cenopalpus umbellatus* sp. nov. Female, (A) dorsum, (B) venter, (C) spermatheca, (D) palp. (Image credit: Mohamed Waleed Negm).

**Figure 2:**
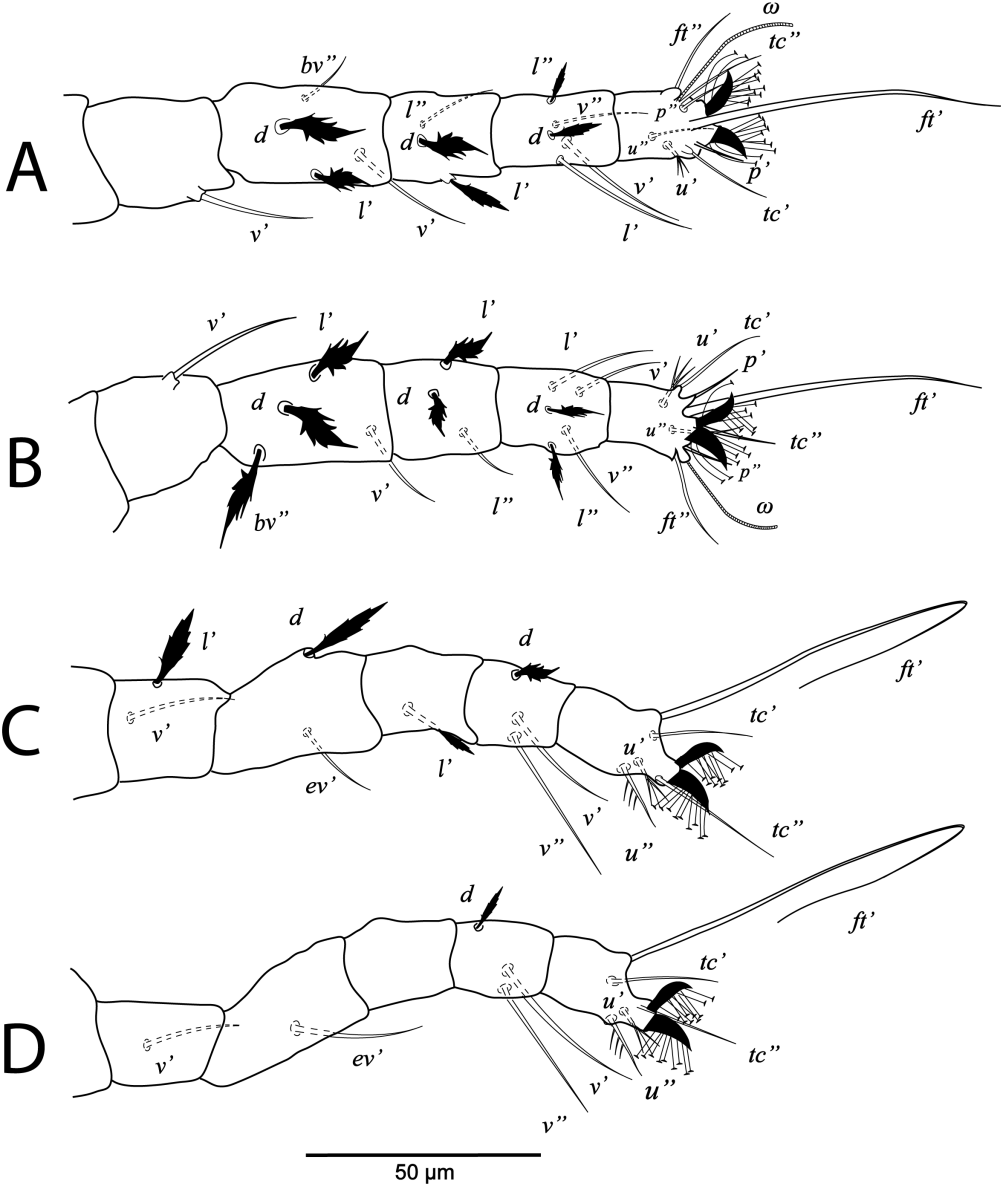
*Cenopalpus umbellatus* sp. nov. Female, (A) leg I (left), (B) leg II (right), (C) leg III (right), (D) leg IV (right). (Image credit: Mohamed Waleed Negm).

**Figure 3:**
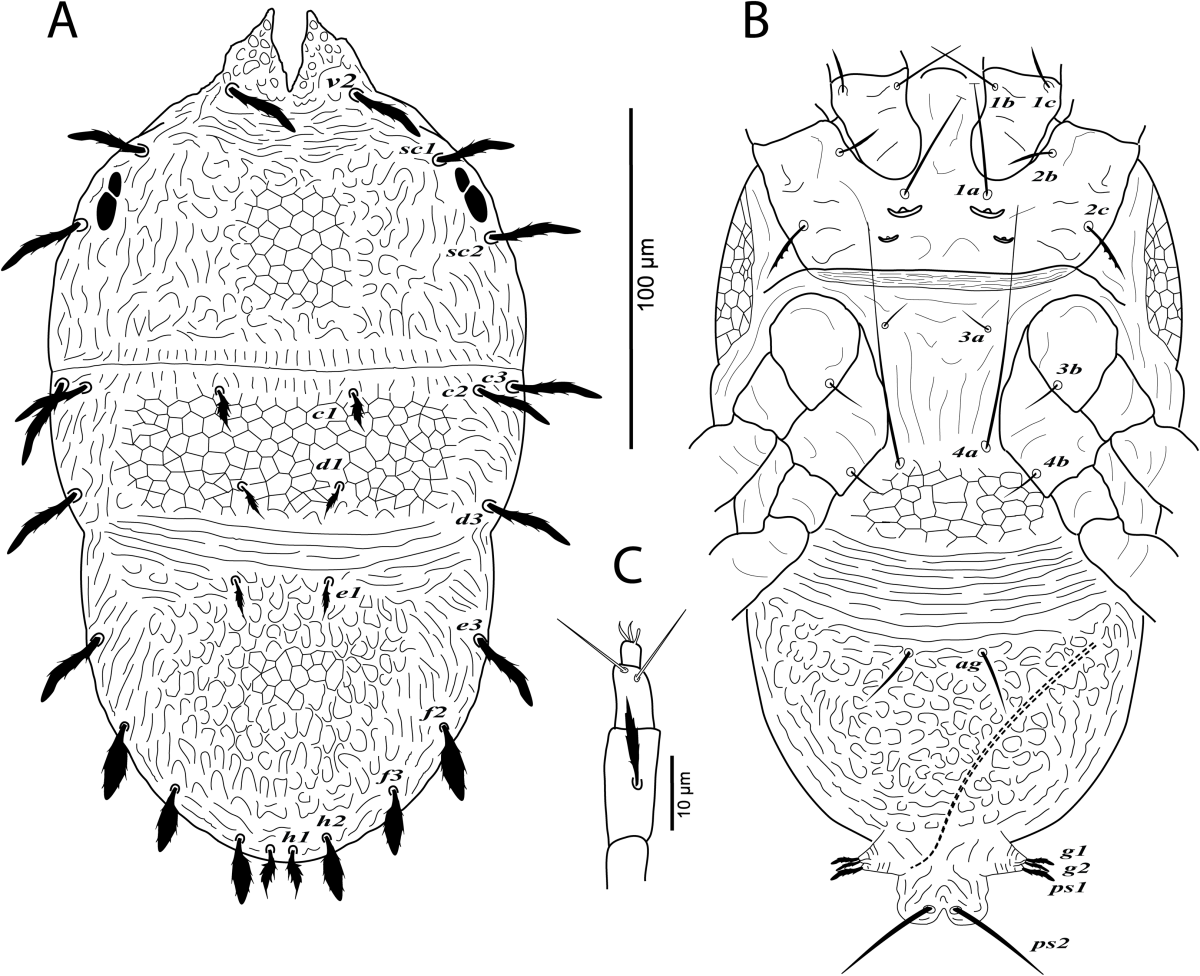
*Cenopalpus umbellatus* sp. nov. Male, (A) dorsum, (B) venter, (C) palp. (Image credit: Mohamed Waleed Negm).

**Figure 4:**
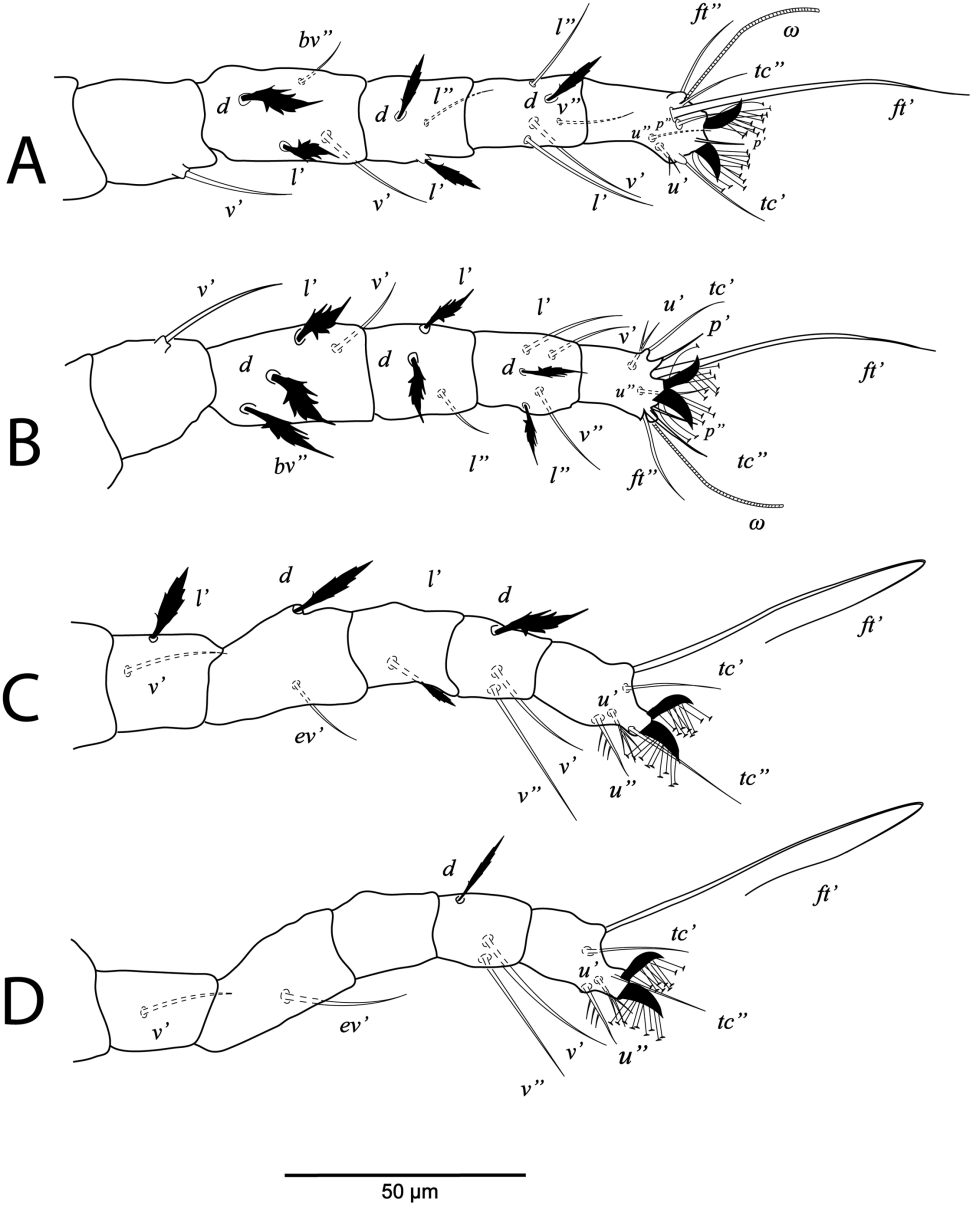
*Cenopalpus umbellatus* sp. nov. Male, (A) leg I (left), (B) leg II (right), (C) leg III (right), (D) leg IV (right). (Image credit: Mohamed Waleed Negm).

**Figure 5:**
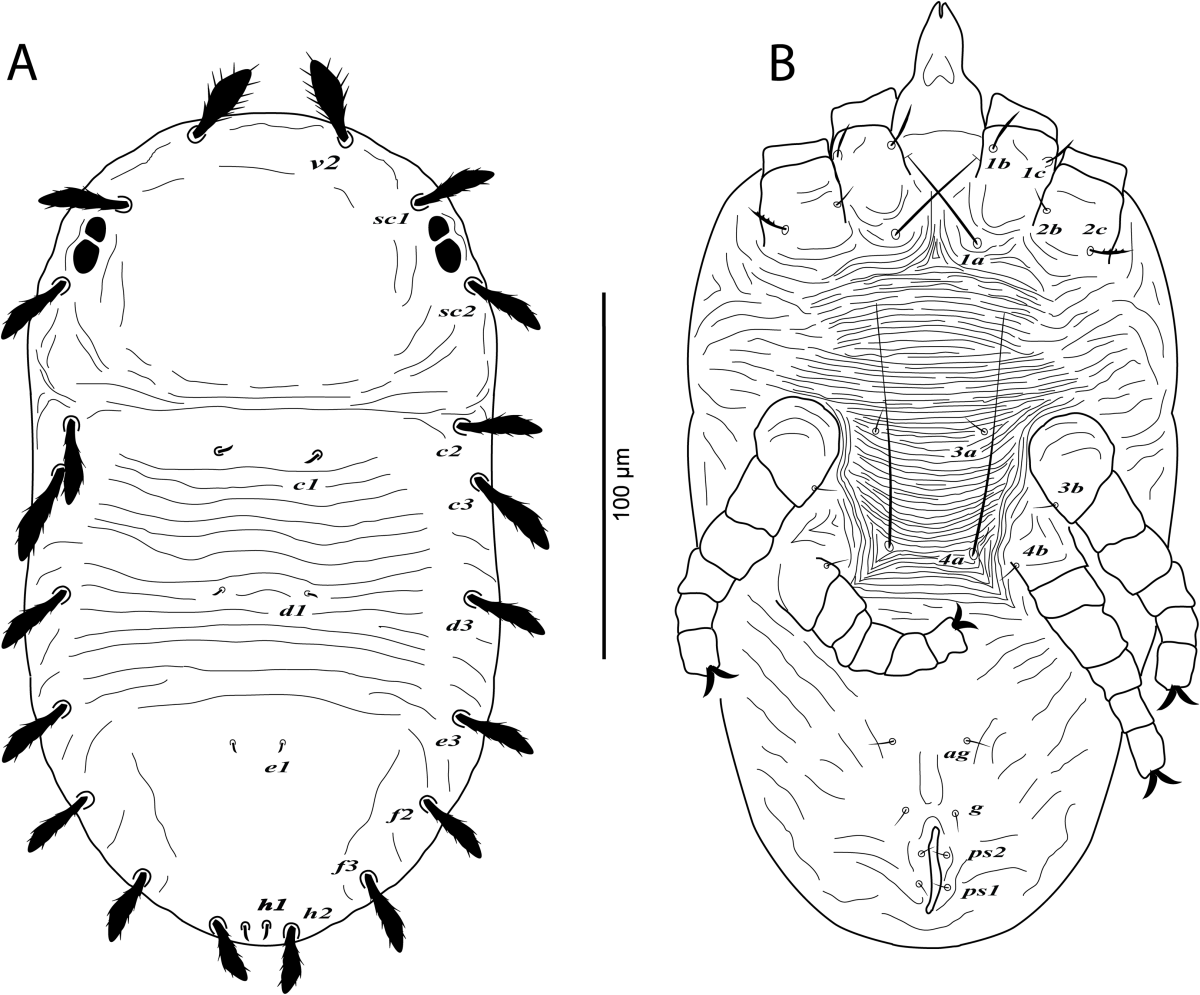
*Cenopalpus umbellatus* sp. nov. Deutonymph, (A) dorsum, (B) venter. (Image credit: Mohamed Waleed Negm).

**Figure 6:**
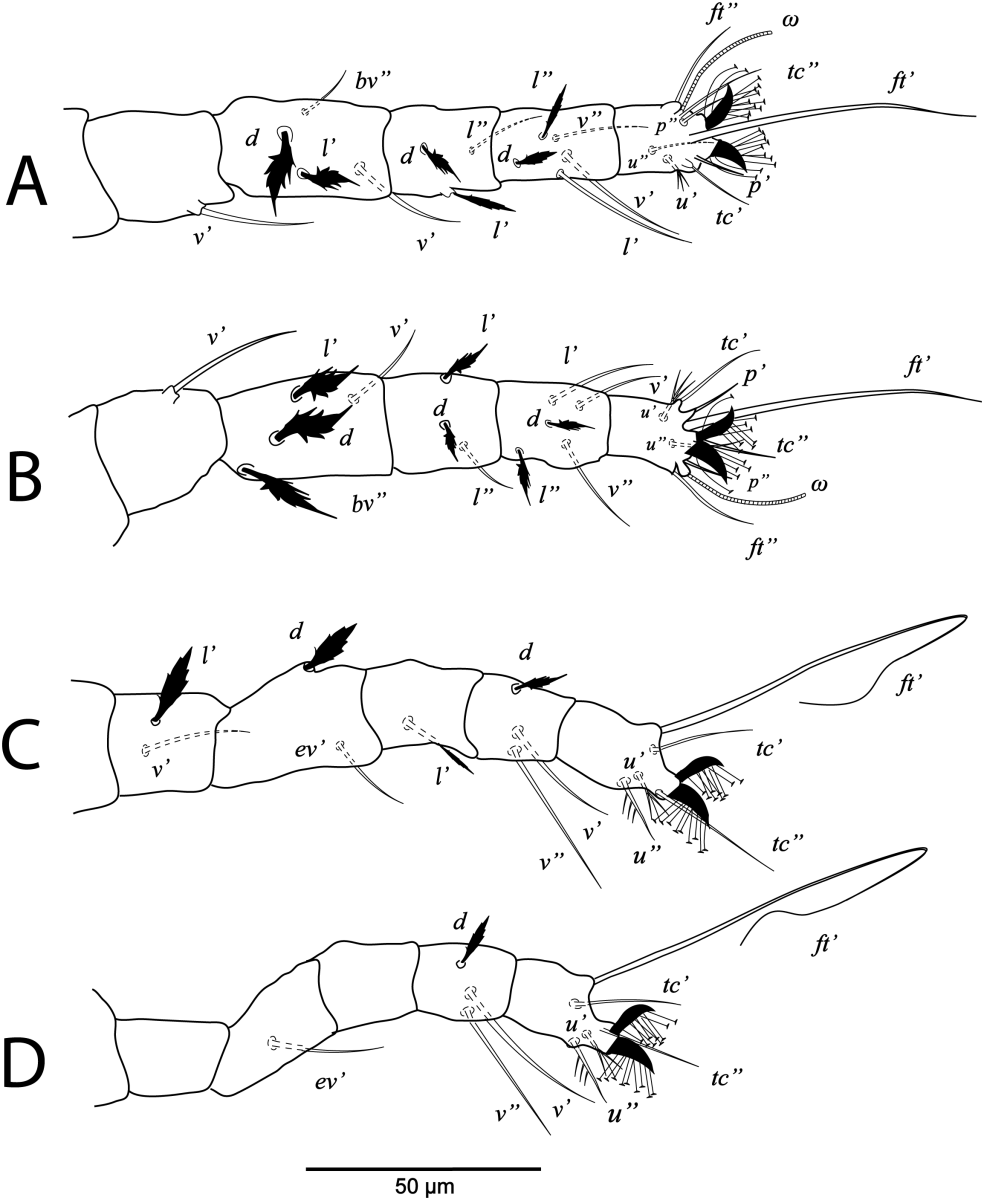
*Cenopalpus umbellatus* sp. nov. Deutonymph, (A) leg I (left), (B) leg II (right), (C) leg III (right), (D) leg IV (right). (Image credit: Mohamed Waleed Negm).

**Figure 7:**
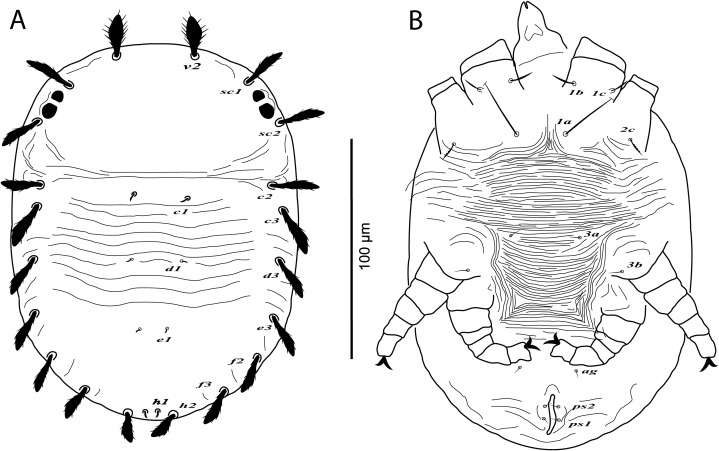
*Cenopalpus umbellatus* sp. nov. Protonymph, (A) dorsum, (B) venter. (Image credit: Mohamed Waleed Negm).

**Figure 8:**
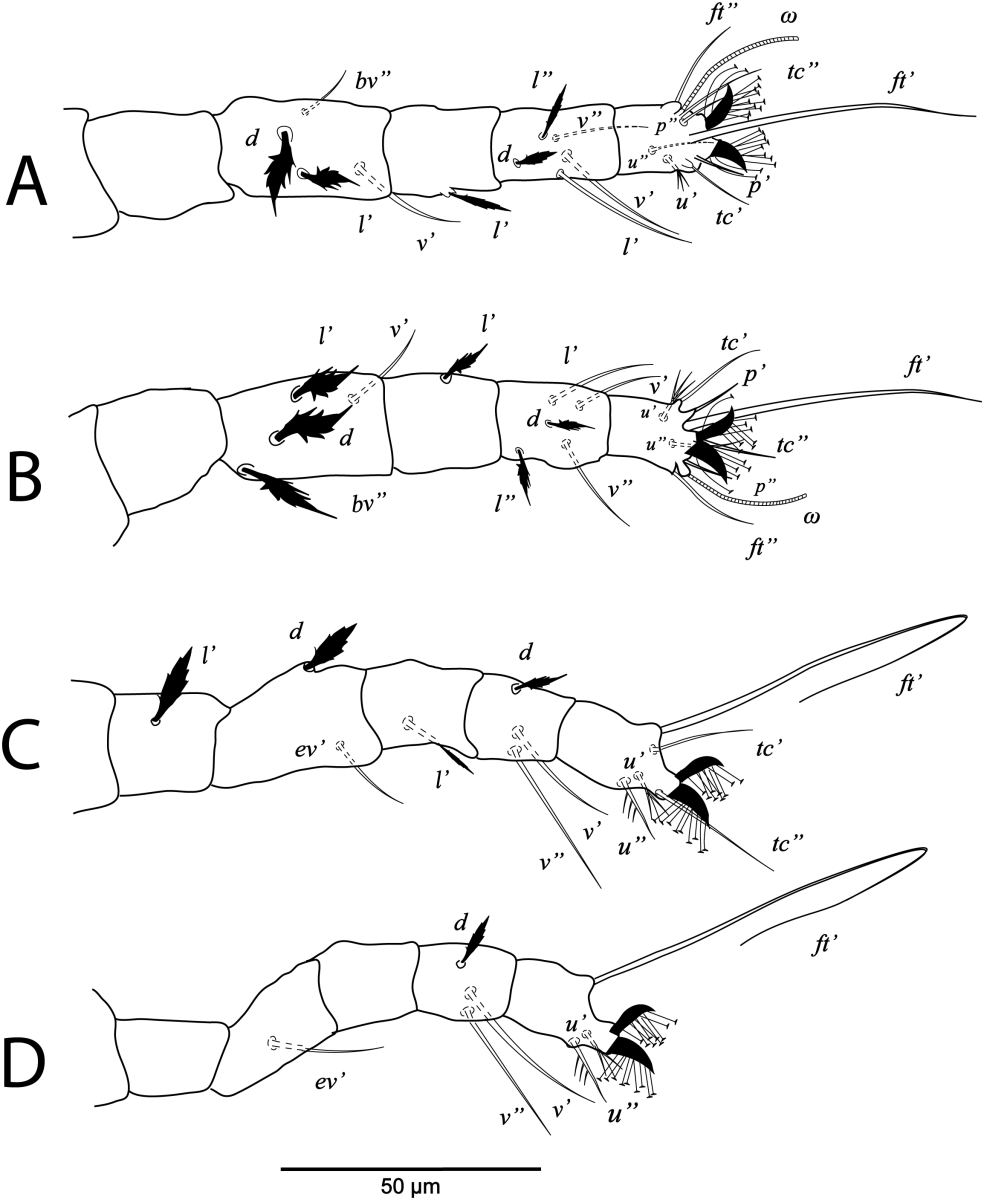
*Cenopalpus umbellatus* sp. nov. Protonymph, (A) leg I (left), (B) leg II (right), (C) leg III (right), (D) leg IV (right). (Image credit: Mohamed Waleed Negm).

**Figure 9:**
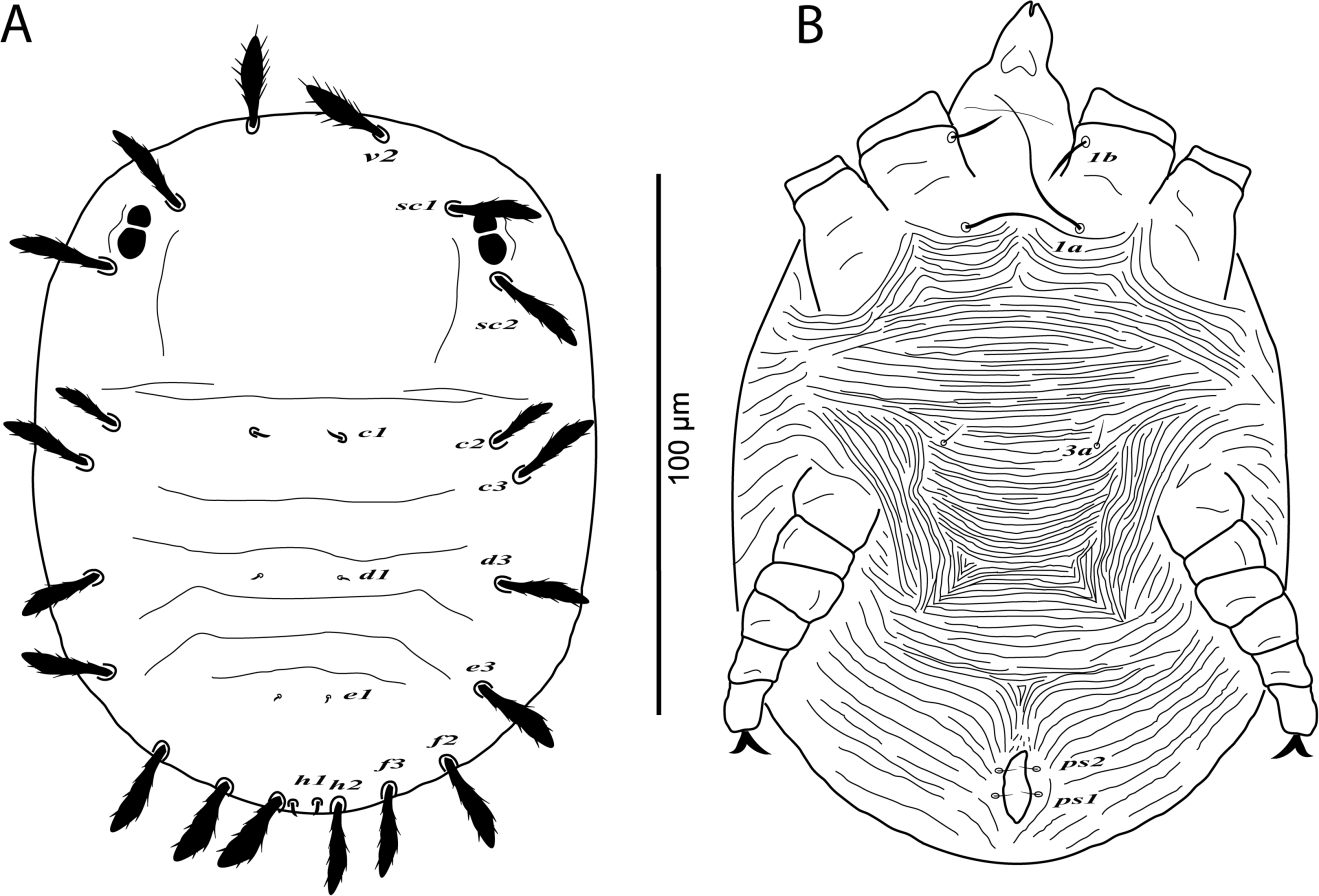
*Cenopalpus umbellatus* sp. nov. Larva, (A) dorsum, (B) venter. (Image credit: Mohamed Waleed Negm).

**Figure 10:**
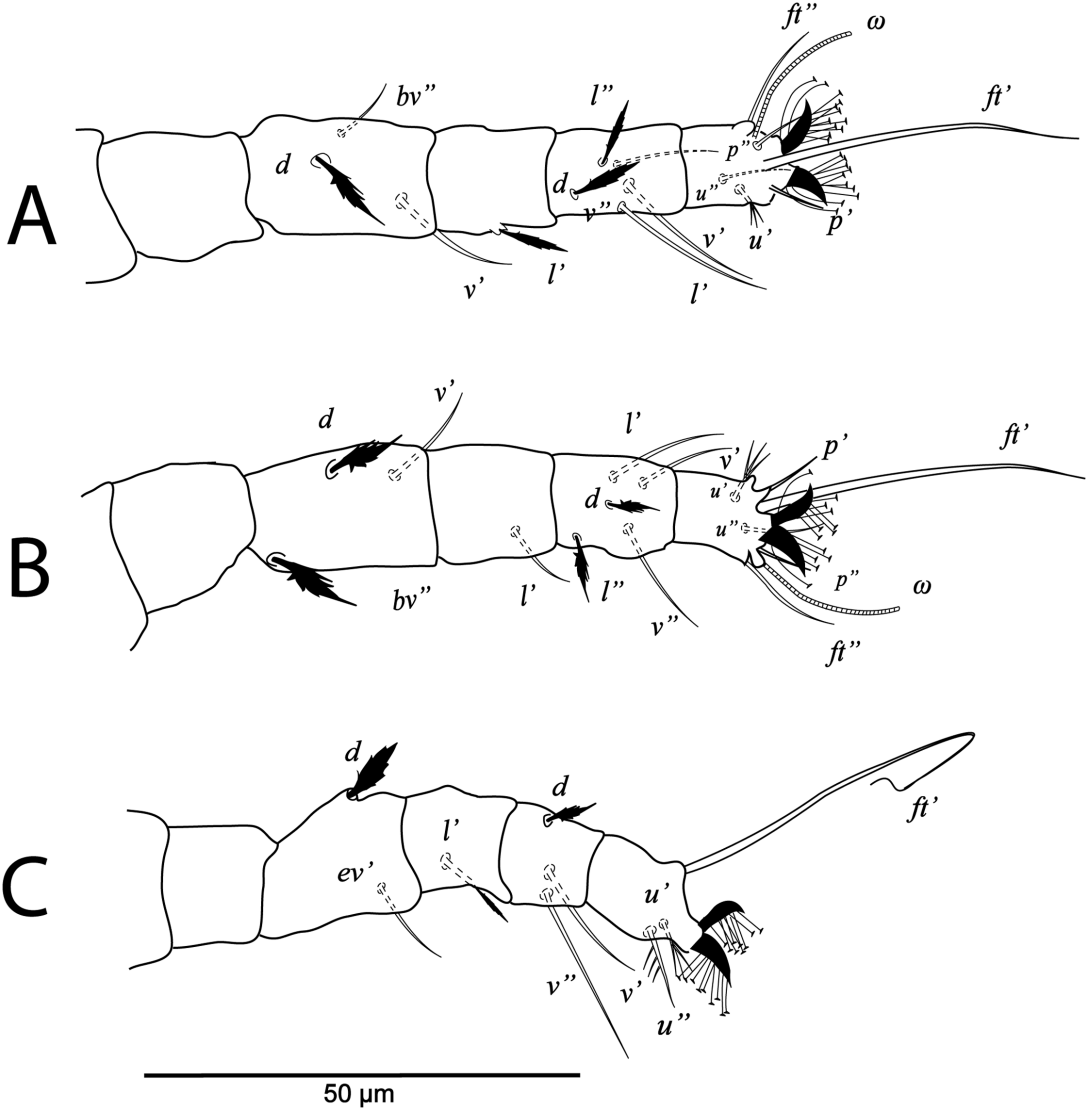
*Cenopalpus umbellatus* sp. nov. Larva, (A) leg I (left), (B) leg II (right), (C) leg III (right). (Image credit: Mohamed Waleed Negm).

## Description

**Female** (*n* = 10)

**Dorsum** (Fig. 1A). Idiosoma oval, length 300 (278–315), excluding gnathosoma; width 170 (157–174), at level of sejugal furrow. Rostral shield with 2 medial, 2 submedial and 2 lateral lobes; propodosoma regularly reticulated, with few irregular areolae sculpturing laterally; sejugal furrow thick and well defined; opisthosoma mostly reticulated, with few irregular transverse reticulations medially and small irregular areolae laterally; opisthosomal pores absent; propodosomal setae *v2* and *sc1* broadly lanceolate, serrate, setae *sc2* narrowly lanceolate; setae *v2* shorter than distance between *v2–v2*; opisthosomal setae narrowly lanceolate. Lengths of dorsal setae: *v2* 24 (22–26), *sc1* 16 (15–17), *sc2* 13 (12–14), *c1* 9 (9–11), *c2* 13 (14–15), *c3* 17 (16–19), *d1* 8 (7–8), *d3* 14 (13–14), *e1* 7 (6–7), *e3* 13 (12–14), *f2* 12 (10–11), *f3* 11 (11–12), *h1* 6 (6–7), *h2* 10 (9–10).

**Venter** (Fig. 1B). Venter of propodosoma and area between setae *3a* and *4a* smooth; opisthosomal area behind ventral setae *4a* entirely reticulated; coxal seta *2c* serrate. ventral shield medially with a reticulation consisting of pentagonal cells; genital shields reticulated with pentagonal cells; genital setae *g1* posterior to *g2*. Lengths of ventral setae: *1a* 80 (75–82), *3a* 9 (8–10), *4a* 70 (65–70); aggenital setae *ag* 13 (12–14); genital setae *g1* 10 (10–12), *g2* 9 (9–11); anal setae *ps1* 10 (9–10), *ps2* 8 (8–10). Distances between genital area setae: *ag–ag* 12–18, *g1–g1* 21–28, *g2–g2* 34–40. Spermatheca (*n* = 3) (Fig. 1C). Spermathecal tube narrow and vesicle semi-circular 8 (8–9) in diameter.

**Gnathosoma**. Rostrum not reaching distal end of femur I. Palp 4-segmented, palp tarsus with a solenidion and 2 eupathidia, palp tibia with 2 setae, palp femur/genu with 1 lanceolate-serrate dorsal seta (Fig. 1D).

**Legs** (Figs. 1B and 2A–2D). Chaetotaxy of legs as follows: coxae 2-2-1-1; trochanters 1-1-2-1; femora 4-4-2-1; genua 3-3-1-0; tibiae 5-5-3-3; tarsi 8+*ω*-8+*ω*-5-5. Setae *d* on femora I-III and genua I-II, setae *l’* on femora I-II and genua I-II broadly lanceolate-serrate. Setae *bv”* on femur II and *l’* on trochanter III also broadly lanceolate-serrate. Tarsus I and II with solenidia I*ω* 15–25, II*ω* 12–18.

**Male** (*n* = 10)

**Dorsum** (Fig. 3A). Idiosoma broadly oval, length 223–238; width 130–140. Rostral shield with 2 medial and 2 slightly shorter submedial lobes; propodosoma regularly reticulated medially, with irregular areolae sculpturing laterally; sejugal furrow distinct; metapodosoma and opisthosoma separated by transverse bands of striae, with irregular reticulations and areolae sculpturing; opisthosomal pores indistinct; propodosomal and lateral setae of opisthosoma long and narrowly lanceolate, serrate; setae *v2* shorter than distance between *v2–v2*. Lengths of dorsal setae: *v2* 27–28, *sc1* 24–26, *sc2* 22–24, *c1* 12–14, *c2* 16–18, *c3* 21–23, *d1* 9–10, *d3* 23–26, *e1* 9–11, *e3* 23–25, *f2* 21–24, *f3* 19–22, *h1* 10–11, *h2* 19–21.

**Venter** (Fig. 3B). Venter of propodosoma and area between setae *3a* and *4a* slightly striated; opisthosomal area behind ventral setae *4a* reticulated, followed by transverse striae posteriorly; coxal seta *2c* serrate; ventral shield posterior to setae *ag* areolate. Lengths of ventral setae: *1a* 58–68, *3a* 10–12, *4a* 55–63; *ag* 18–20; *g1* 8–9, *g2* 9–10; *ps1* 10–12, *ps2* 26–28.

**Gnathosoma**. Rostrum short not reaching distal end of trochanter I. Palp 4-segmented, palp tarsus with a solenidion and 2 eupathidia, palp tibia with 2 setae, palp femur/genu with 1 lanceolate-serrate dorsal seta (Fig. 3C).

**Legs** (Figs. 3B and 4A–4D). Chaetotaxy of legs as in female. Leg setae also similar to that of female. Tarsus I and II with solenidia I*ω* 25–30, II*ω* 20–23.

**Deutonymph** (*n* = 6)

**Dorsum** (Fig. 5A). Idiosoma oval, length 257–266; width 144–162. Rostral shield absent; propodosoma rounded anteriorly, smooth; opisthosoma with transverse striae in the area between setae *c1* and *e1*; opisthosomal pores absent. Dorsal body setae long and narrowly lanceolate except dorsocentral setae *c1*, *d1*, *e1*, *h1* minute; setae *v2* distinctly shorter than distance between *v2*–*v2*. Lengths of dorsal setae: *v2* 28–30, *sc1* 26–27, *sc2* 25–27, *c1* 4–6, *c2* 23–25, *c3* 25–27, *d1* 2–4, *d3* 23–25, *e1* 2–3, *e3* 22–24, *f2* 21–23, *f3* 20–22, *h1* 4–6, *h2* 16–18.

**Venter** (Fig. 5B). Venter of propodosoma and area between setae *1a* and *4a* with transverse striae; seta *2c* serrate; posterior opisthosomal area with irregular striae. Lengths of ventral setae: *1a* 42–48, *3a* 6–8, *4a* 38–45; *ag* 6–7; *g1* 4–5; *ps1* 3–4, *ps2* 3–4.

**Gnathosoma**. Palp 4-segmented, palp chaetotaxy as in female.

**Legs** (Figs. 5B and 6A–6D). Chaetotaxy of legs: coxae 2-2-1-1; trochanters 1-1-2-0; femora 4-4-2-1; genua 3-3-1-0; tibiae 5-5-3-3; tarsi 8+*ω*-8+*ω*-5-5. Leg setae similar to that of female.

**Protonymph** (*n* = 2)

**Dorsum** (Fig. 7A). Idiosoma broadly oval, length 164–170; width 106–110. Rostral shield absent; propodosoma rounded anteriorly, smooth; opisthosoma with transverse striae in the area between setae *c1* and *e1*; opisthosomal pores absent. Dorsal body setae long and narrowly lanceolate except dorsocentral setae *c1*, *d1*, *e1*, *h1* minute; setae *v2* distinctly shorter than distance between *v2*–*v2*. Lengths of dorsal setae: *v2* 21–24, *sc1* 17–18, *sc2* 19–21, *c1* 4–5, *c2* 16–18, *c3* 19–20, *d1* 2–3, *d3* 17–19, *e1* 2–3, *e3* 14–15, *f2* 15–17, *f3* 15–16, *h1* 3–5, *h2* 12–13.

**Venter** (Fig. 7B). Venter of idiosoma with transverse striae; posterior opisthosomal area with irregular striae; seta *2c* smooth or slightly serrate, *2b* absent; ventral setae *4a*, *4b* and genital setae *g* absent. Lengths of ventral setae: *1a* 31–40, *3a* 4–5; *ag* 3–4; *ps1* 2–3, *ps2* 2–3.

**Gnathosoma**. Palp 4-segmented, palp chaetotaxy as in deutonymph.

**Legs** (Figs. 7B and 8A–8D). Chaetotaxy of legs: coxae 2-1-1-0; trochanters 0-0-1-0; femora 4-4-2-1; genua 1-1-1-0; tibiae 5-5-3-3; tarsi 8+*ω*-8+*ω*-5-3. Leg setae similar to that of female.

**Larva** (*n* = 4)

**Dorsum** (Fig. 9A). Idiosoma broadly oval, length 150–162; width 110–118. Rostral shield absent; idiosoma smooth, with few transverse striae posteriorly; opisthosomal pores absent. Dorsal body setae long and narrowly lanceolate except dorsocentral setae *c1*, *d1*, *e1*, *h1* minute; setae *v2* shorter than distance between *v2*–*v2*. Lengths of dorsal setae: *v2* 16–18, *sc1* 14–16, *sc2* 15–17, *c1* 3–4, *c2* 12–14, *c3* 15–16, *d1* 2–3, *d3* 15–17, *e1* 2–3, *e3* 17–18, *f2* 16–17, *f3* 16–17, *h1* 3–5, *h2* 17–18.

**Venter** (Fig. 9B). Venter of idiosoma completely striated; ventral setae *4a*, coxal setae *1c*, *2b*, *2c*, *3b*, aggenital setae *ag* and genital setae *g* absent. Lengths of ventral setae: *1a* 28–34, *3a* 6–7; *ps1* 3–4, *ps2* 2–3.

**Gnathosoma**. Palp 4-segmented, palp chaetotaxy as in female.

**Legs** (Figs. 9B and 10A–10C). Chaetotaxy of legs: coxae 1-0-0; trochanters 0-0-0; femora 3-3-2; genua 1-1-1; tibiae 5-5-3; tarsi 6+*ω*-6+*ω*-3.

**Type material**

Female holotype, 24 female paratypes, 10 male paratypes, six deutonymphs, two protonymphs and four larvae; ex. leaves of *Rhaphiolepis indica* var. *umbellata* Makino (Rosaceae); Chiba, Japan (35°02′16″N, 139°50′15″E); 14 June 2018; leg. M.W. Negm. Type depository: female holotype, two female paratypes, three male paratypes, two deutonymphs, two protonymphs and two larvae will be deposited in the National Museum of Nature and Science (NMNS), Tsukuba, Ibaraki Prefecture, Japan. The remainder types are deposited in the Laboratory of Applied Entomology and Zoology, Ibaraki University (AEZIU) with the voucher specimen no. 895.

**Etymology**

The specific name *umbellatus* is named after the host plant species. The gender is masculine.

**Differential diagnosis**

*Cenopalpus umbellatus* sp. nov. closely resembles *C. lanceolatisetae* (Attiah, 1956) in various aspects including the chaetotaxy of legs; however, female differs in having rostrum not reaching distal end of femur I (vs. rostrum extending to middle of genu I in *C. lanceolatisetae*), reticulations behind ventral setae *4a* medially connected (vs. smooth or slightly striate medially in *C. lanceolatisetae*) and variation in lengths of some idiosomal setae (Table 3). Male of *C. umbellatus* sp. nov. also differs in having reticulations behind ventral setae *4a* (vs. reticulations absent in *C. lanceolatisetae*) and in having no opisthosomal pores (vs. one pair of opisthosomal pores present in *C. lanceolatisetae*). Also, the deutonymph of the new species has propodosoma smooth medially (vs. propodosoma reticulated medially in *C. lanceolatisetae*).

**Table 3:** Measurements of idiosomal setae for *Cenopalpus umbellatus* sp. nov. and its congener *C. lanceolatisetae* (Attiah, 1956).

Setae	*C. lanceolatisetae* (range for 10 females) (Khanjani et al., 2012)	*C. umbellatus* sp. nov. holotype (range for paratypes)
*v2*	18–26	24 (22–26)
*sc1*	17–23	16 (15–17)
*sc2*	18–24	13 (12–14)
*c1*	11–16	9 (9–11)
*c2*	13–19	13 (14–15)
*c3*	12–18	17 (16–19)
*d1*	7–11	8 (7–8)
*d3*	11–18	14 (13–14)
*e1*	7–12	7 (6–7)
*e3*	13–16	13 (12–14)
*f2*	13–16	12 (10–11)
*f3*	10–14	11 (11–12)
*h1*	5–9	6 (6–7)
*h2*	10–14	10 (9–10)
*1a*	75–103	80 (75–82)
*3a*	12–16	9 (8–10)
*4a*	80–119	70 (65–70)
*ag*	13–18	13 (12–14)
*g1*	9–12	10 (10–12)
*g2*	8–13	9 (9–11)
*ps1*	12–16	10 (9–10)
*ps2*	5–10	8 (8–10)

**Ontogeny**

The ontogenetic changes in the idiosomal and leg chaetotaxy of *Cenopalpus umbellatus* sp. nov. resemble the typical pattern for tenuipalpid mites (Lindquist, 1985). Regarding the setal additions on ventral idiosoma, the ventral (*1a*, *3a*) and anal (*ps2*, *ps1*) setae appeared since the larval stage. However, aggenital seta (*ag*) is added in the protonymph and the ventral seta (*4a*) is added in the deutonymph. Also, genital setae (*g1*) appeared in the deutonymph and *g2* in the adults. The coxal setae *1c*, *2c* and *3b* are added in the protonymph and the setae *2b* and *4b* are added in the deutonymph. Setae *v’* appeared on trochanters I, II and III in the deutonymph while appeared on trochanters IV in the adults. Seta *l’* on trochanter III is added in the protonymph. Also, seta *l’* is added to femora I and II in protonymph. Setae *l’* is present on genua I and II of the larva. Setae *d* and *l”* are added to genua I and II in the deutonymph. The tectal setae (*tc’*, *tc”*) are added to tarsus I, II and III in the protonymphal stage.

**Key to world species of *Cenopalpus* (based on females)**

1. Opisthosoma with 6 pairs of dorsolateral setae2

—Opisthosoma with 7 pairs of dorsolateral setae7

2. Palp-tibia and palp-tarsus with 2 setae each3

—Palp-tibia with 1 seta and palp-tarsus with 2 setae*creticus*

3. Rostrum extending beyond distal end of femur I4

—Rostrum extending to mid-level of femur I, not reaching to distal end5

4. Dorsal setae rod-like*pistaciae*

—Dorsal setae feather-like*pterinus*

5. Setal formula of tibiae 5-5-3-36

—Setal formula of tibiae 5-5-5-3*arbuti*

6. Setal formula of trochanters 1-1-1-1; reticulations behind setae *4a* partly separated medially*officinalis*

—Setal formula of trochanters 1-1-2-1; reticulations behind setae *4a* prominent and not separated medially*adventicius*

7. Idiosoma mostly striate or partly striate and partly reticulate8

—Idiosoma mostly reticulate12

8. Dorsum mostly striate but also with reticulations on prodorsum and between *c* and *d* series on hysterosoma; setae *3a* and *4a* very long*tamarixi*

—Dorsum striate with setae *4a* much longer than short *3a*9

9. Rostral shield with 2 slightly notched medial lobes10

—Rostral shield with 2 medial and 2 lateral lobes11

10. Setae *4a* on venter much longer than distance between setae *3a* and *4a*, setae *1a* very long and whip-like extending considerably pass rostrum*wainsteini*

—Setae *4a* approximately equal to, or little longer than, distance between setae *3a* and *4a*, setae *1a* not extending pass rostrum*saryabiensis*

11. Rostrum reach almost to middle of genu I; hysterosoma with transverse striae from prodorsum to behind setae *d1* and longitudinal to posterior margin*aratus*

—Rostrum reach almost to middle of femur I; striae on hysterosoma mainly transverse*lineola*

12. Propodosomal setae broadly lanceolate to spatulate or scoop-like13

—Propodosomal setae narrowly lanceolate to setiform or slender37

13. Propodosomal setae broadly lanceolate to spatulate; opisthosomal pores absent (one pair present in *pennatisetis*)14

—Propodosomal setae scoop-like; 2 pairs of opisthosomal pores present*scoopsetus*

14. Rostrum reaching behind distal end of femur I15

—Rostrum not reaching beyond distal end of femur I30

15. Rostrum extending beyond distal end of genu I16

—Rostrum not extending beyond distal end of genu I18

16. Setae *sc1* shorter than distance between bases of setae *sc1* and *sc2*17

—Setae *sc1* longer than distance between bases of setae *sc1* and *sc2**khosrowshahi*

17. Setae *sc1* less than half of distance between bases of setae *sc1* and *sc2**prunusi*

—Setae *sc1* more than half of distance between bases of setae *sc1* and *sc2**longirostris*

18. Propodosoma with reticulations regular19

—Propodosoma with reticulations irregular26

19. Setae *sc1* shorter than distance between bases of setae *sc1* and *sc2.*20

—Setae *sc1* longer than, or equal to, distance between bases of setae *sc1* and *sc2*23

20. Dorsal body setae subspatulate, narrowly or broadly lanceolate21

—Dorsal body setae broadly spatulate*eriobotryi*

21. Setae *v2* broadly lanceolate and much longer than half of distance between their bases; rostral shield with 2 medial, 2 submedial and 2 lateral lobes22

—Setae *v2* narrowly lanceolate and equal to, or little longer than, half of distance between their bases; rostral shield with 2 medial lobes*chitraliensis*

22. Metapodosomal venter posterior to setae *4a* smooth medially or slightly striate; rostrum extending to middle of genu I*lanceolatisetae*

—Metapodosomal reticulations on venter posterior to setae *4a* connected medially; rostrum not reaching pass distal end of femur I*umbellatus* sp. nov.

23. Dorsal setae subspatulate with long spines*viniferus*

—Dorsal setae subspatulate or narrowly lanceolate and serrate24

24. Dorsal setae narrowly lanceolate and setae *c1* almost as long as distance between its members25

—Dorsal setae subspatulate with setae *c1* clearly shorter than distance between its members*xini*

25. Setal formula of trochanters 1-1-2-1, femora 4-4-2-1*pennatisetis*

—Setal formula of trochanters 1-1-1-1, femora 4-4-2-0*virgulatus*

26. Setae *v2* shorter than distance between their bases27

—Setae *v2* longer than, or equal to, distance between their bases28

27. Rostrum at level of distal end of genu I; rostral shield basically with only 2 medial lobes*halperini*

—Rostrum not reaching distal end of genu I; rostral shield with 2 medial and 2 lateral lobes*pegazzanoae*

28. Rostrum reaching to middle or to distal margin of genu I; propodosomal setae broadly lanceolate29

—Rostrum reaching beyond distal end of femur I; propodosomal setae spatulate*evini*

29. Propodosoma with large polygonal reticulations medially*abaii*

—Propodosoma smooth or weakly reticulate medially*bagdasariani*

30. Dorsal body setae spatulate or subspatulate31

—Dorsal body setae lanceolate*haqii*

31. Dorsal body setae spatulate32

—Dorsal body setae subspatulate34

32. Propodosoma with regular polygonal reticulations*capensis*

—Propodosoma with irregular reticulations, especially mediodorsally and mediolaterally33

33. Metapodosomal venter with area posterior to setae *4a* completely reticulated, anterior to *4a* weakly reticulate*salignae*

—Metapodosomal venter with area posterior to setae *4a* smooth medially or slightly striate and smooth anterior to *4a**oleunus*

34. Metapodosomal venter with area posterior to setae *4a* smooth medially35

—Metapodosomal venter with area posterior to setae *4a* reticulated36

35. Setae *v2* equal to, or little shorter than, distance between their bases*platani*

—Setae *v2* approximately half of distance between their bases*ramus*

36. Setae *v2* approximately half of distance between their bases; idiosoma with dorsal reticulations regular; dorsal setae short and serrate*natalensis*

—Setae *v2* equal to distance between their bases; idiosoma with dorsal reticulations irregular; dorsal setae clearly longer and strongly serrate*pritchardi*

37. Setae *v2* approximately longer than, or equal to, distance between their bases38

—Setae *v2* shorter than distance between their bases51

38. Rostral shield with 2 medial lobes, lateral lobes excluded39

—Rostral shield with 4 medial lobes, one pair can be reduced or obsolete, lateral lobes also excluded42

39. Rostrum reaching up to distal end of femur I; metapodosomal venter with area posterior to setae *4a* smooth medially40

—Rostrum reaching to middle of genu I; metapodosomal venter with area posterior to setae *4a* reticulated41

40. Setal formula of tibiae 4-4-3-3*mughalii*

—Setal formula of tibiae 5-5-3-3*orakiensis*

41. Propodosoma with small, rounded crenulate elements*spinosus*

—Propodosoma with large polygonal reticulations*pulcher*

42. Dorsal body setae mostly lanceolate43

—Dorsal body setae mostly setiform47

43. Opisthosoma with pores44

—Opisthosoma without pores45

44. Rostrum not extending beyond distal end of femur I, rostral shield with 4 distinct lobes medially*quadricornis*

—Rostrum extending beyond distal end of femur I, second pair of medial lobes obsolete*irani*

45. Setae *c1* and *d1* long, almost as long as distances between their members*quercusi*

—Setae *c1* and *d1* much shorter, half or less than half the distances between their members.46

46. Setal formula of genua 3-3-3-1, trochanters 1-1-2-1*taygeticus*

—Setal formula of genua 3-3-1-0, trochanters 1-1-1-1*naupakticus*

47. Setae *sc1* approximately equal to, or longer than, distance between bases of setae *sc1* and *sc2*48

—Setae *sc1* distinctly shorter than distance between bases of setae *sc1* and *sc2**meyerae*

48. Setae *sc1* approximately equal to distance between bases of setae *sc1* and *sc2*49

—Setae *sc1* distinctly longer than distance between bases of setae *sc1* and *sc2**brachypalpus*

49. Setae *sc2* long, almost reaching to sejugal furrow*musai*

—Setae *sc2* short, distinctly far from sejugal furrow50

50. Venter between setae *3a* and *4a* striate*rubusi*

—Venter between setae *3a* and *4a* smooth*pseudospinosus*

51. Rostrum extending to middle of femur I or somewhat beyond middle52

—Rostrum extending to distal end of femur I or beyond56

52. Opisthosoma with dorsolateral setae *c3* about a fifth as long as distance to bases of setae *d3*53

—Opisthosoma with dorsolateral setae *c3* about a third as long as distance to bases of setae *d3*54

53. Setae *v2* shorter than half of distance between their bases; reticulations ventrally behind setae *4a* continuous*cumanicus*

—Setae *v2* longer than half of distance between their bases; reticulations behind setae *4a* smooth medially*thelycraniae*

54. Metapodosomal venter at area posterior to setae *4a* with smaller polygonal to rounded crenulate elements medially55

—Metapodosomal venter at area posterior to setae *4a* with medial reticulation elements polygonal and broader than long*carpini*

55. Setae *v2* shorter than half of distance between their bases*hederae*

—Setae *v2* longer than half of distance between their bases*mespili*

56. Rostrum reaching not beyond distal end of genu I; palp-tarsus with at least a solenidion and seta or eupathium57

—Rostrum reaching to distal end of tibia I; palp-tarsus with 1 solenidion only*picitilis*

57. Rostrum reaching to mid-level or distal end of genu I58

—Rostrum reaching not beyond distal end of femur I63

58. Dorsal setae narrowly lanceolate59

—Dorsal setae setiform60

59. Body almost round; rostrum reaching distal end of genu I; setal formula of tibiae 4-4-3-3*sunniensis*

—Body oval; rostrum reaching to mid-level of genu I, not reaching distal end; setal formula of tibiae 5-5-3-3*ruber*

60. All dorsal setae serrate61

—All dorsal setae simple*dignus*

61. Rostrum reaching distal end of femur I62

—Rostrum reaching distal end of genu I*favosus*

62. Setae *v2* more than 15 µm length; setal formula of tibiae 5-4-3-3, coxae 2-2-1-1*kritos*

—Setae *v2* less than 10 µm; setal formula of tibiae 5-5-3-3, coxae 3-2-1-1*homalos*

63. Rostral shield with 2 medial lobes64

—Rostral shield with more than 2 lobes65

64. Metapodosoma with large polygonal reticulation medioventrally; setae *4a* much longer than distance between bases of setae *3a* and *4a*; setal formula of coxae 2-2-1-1, trochanters 1-1-2-2*piger*

—Metapodosoma with irregular reticulation medioventrally; setae *4a* shorter than distance between bases of setae *3a* and *4a*; setal formula of coxae 2-2-2-1, trochanters 1-1-1-0*japonicus*

65. Reticulations almost absent or medially smooth behind ventral setae *4a*66

—Area behind setae *4a* completely reticulated68

66. Area behind setae *4a* almost smooth with only a few reticulations behind coxae IV; dorsal setae narrowly lanceolate and serrate or short setiform, serrate67

—Reticulations behind setae *4a* with a narrow smooth band medially; dorsal setae short, setiform and serrate or some smooth*iqbali*

67. Dorsal setae narrowly lanceolate, serrate*capacis*

—Dorsal setae short, setiform, serrate*limbatus*

68. Rostral shield with 2 medial and 2 lateral lobes69

—Rostral shield with 2 medial, 2 submedial and 2 lateral lobes*crataegi*

69. Propodosomal setae narrowly lanceolate; some setae on opisthosoma also lanceolate*populi*

—All dorsal setae setiform*bakeri*

## Discussion

The present study provides morphological description of a new species of flat mites belonging to the genus *Cenopalpus*, with a key to the world species. This genus is mainly reported from the Mediterranean and East Asia regions. Only 14 tenuipalpid species were previously known from Japan, with only one *Cenopalpus* species. Though members of the Tenuipalpidae are currently not posing a serious threat to agriculture in the country, we must be prepared for the consequences of global trafficking of people and goods. Therefore, this study will for sure act as a very useful early intervention tool. Examination of all known species of *Cenopalpus* was toilsome especially with some species which are poorly described, and we had to rely on what was available.

## Conclusions

Faunistic information about flat mites in Japan is scarce. The new mite species described with the world key to species increases the available information about the taxonomy of tenuipalpid mites in this country. We hope that this study will serve as the departure point for future research on *Cenopalpus* mites and encourage for more comprehensive surveys in Japan since a large number of undiscovered species is expected.

## Supplemental Information

10.7717/peerj.9081/supp-1 Supplemental Information 1 Raw data: measurements per individual.

10.7717/peerj.9081/supp-2 Supplemental Information 2 Raw data: Type material, accession no 895 (AEZIU).

## Additional Information and Declarations

### Data Availability

The following information was supplied regarding data availability: The measurements and type materials information are available in the Supplemental Files. The accession numbers as follows: Laboratory of Applied Entomology and Zoology, Ibaraki University (AEZIU): 4th floor, Faculty of Agriculture, Ibaraki University, Ami, Ibaraki 300-0393, Japan. Paratype female (yellow label) > 895-A1 Paratype female (yellow label) > 895-A2 Paratype female (yellow label) > 895-A3 Paratype female (yellow label) > 895-A4 Paratype female (yellow label) > 895-A5 Paratype female (yellow label) > 895-A6 Paratype female (yellow label) > 895-A7 Paratype female (yellow label) > 895-A8 Paratype female (yellow label) > 895-A9 Paratype female (yellow label) > 895-A10 Paratype female (yellow label) > 895-A11 Paratype female (yellow label) > 895-A12 Paratype female (yellow label) > 895-A13 Paratype female (yellow label) > 895-A14 Paratype female (yellow label) > 895-A15 Paratype female (yellow label) > 895-A16 Paratype female (yellow label) > 895-A17 Paratype female (yellow label) > 895-A18 Paratype female (yellow label) > 895-A19 Paratype female (yellow label) > 895-A20 Paratype female (yellow label) > 895-A21 Paratype female (yellow label) > 895-A22 Paratype male (white label) > 895-A23 Paratype male (white label) > 895-A24 Paratype male (white label) > 895-A25 Paratype male (white label) > 895-A26 Paratype male (white label) > 895-A27 Paratype male (white label) > 895-A28 Paratype male (white label) > 895-A29 Deutonymph (white label) > 895-A30 Deutonymph (white label) > 895-A31 Deutonymph (white label) > 895-A32 Deutonymph (white label) > 895-A33 Larva (white label) > 895-A34 Larva (white label) > 895-A35 National Museum of Nature and Science (NMNS): 4 Chome-1-1 Amakubo, Tsukuba, Ibaraki 305-0005, Japan. Holotype female (red label) > 895-B1 Paratype female (yellow label) > 895-B2 Paratype female (yellow label) > 895-B3 Paratype male (white label) > 895-B4 Paratype male (white label) > 895-B5 Paratype male (white label) > 895-B6 Deutonymph (white label) > 895-B7 Deutonymph (white label) > 895-B8 Protonymph (white label) > 895-B9 Protonymph (white label) > 895-B10 Larva (white label) > 895-B11 Larva (white label) > 895-B12.

### New Species Registration

The following information was supplied regarding the registration of a newly described species: Publication LSID: urn:lsid:zoobank.org:pub:268B04C7-028B-4C03-8F6C-930035941B89. Cenopalpus umbellatus sp. nov. LSID: urn:lsid:zoobank.org:act:957E754C-A7F0-4081-A814-48115D276F76.
