# Supplementary figures and images for: A new species of Cenopalpus Pritchard & Baker (Acari: Tenuipalpidae) from Japan, with ontogeny of chaetotaxy and a key to the world species
Source: PeerJ. 2020 Apr 27;8:e9081. doi: 10.7717/peerj.9081 (PMC7199764; doi:10.7717/peerj.9081)

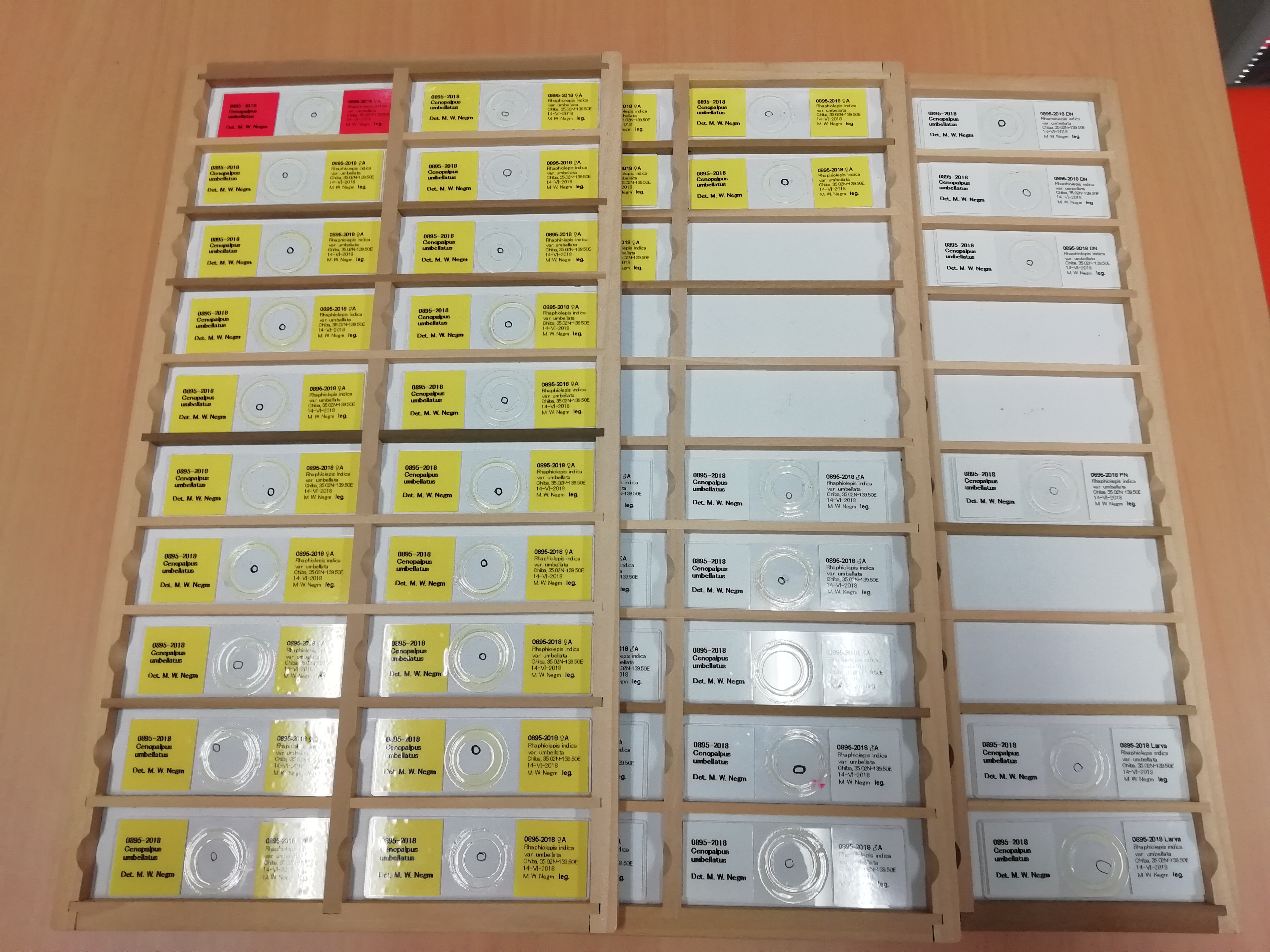

Supplement: Supplemental Information 2 [file peerj-08-9081-s002.jpg]
